# Novel brain expressed RNA identified at the MIR137 schizophrenia-associated locus

**DOI:** 10.1016/j.schres.2016.11.034

**Published:** 2017-06

**Authors:** Olympia Gianfrancesco, Alix Warburton, David A. Collier, Vivien J. Bubb, John P. Quinn

**Affiliations:** a Department of Molecular and Clinical Pharmacology, Institute of Translational Medicine, The University of Liverpool, Liverpool L69 3BX, UK; b Eli Lilly and Company Limited, Windlesham, Surrey, UK

**Keywords:** EU358092, lncRNA, microRNA-137, Schizophrenia

## Abstract

Genome-wide association studies (GWAS) have identified a locus on chromosome 1p21.3 to be highly associated with schizophrenia. A microRNA, MIR137, within this locus has been proposed as the gene causally associated with schizophrenia, due to its known role as a regulator of neuronal development and function. However, the involvement of other genes within this region, including DPYD (dihydropyrimidine dehydrogenase), is also plausible. In this communication, we describe a previously uncharacterised, brain-expressed RNA, EU358092, within the schizophrenia-associated region at 1p21.3. As we observed for MIR137, EU358092 expression was modulated in response to psychoactive drug treatment in the human SH-SY5Y neuroblastoma cell line. Bioinformatic analysis of publically available CNS expression data indicates that MIR137 and EU358092 are often co-expressed in vivo. A potential regulatory domain for expression of EU358092 is identified by bioinformatic analysis and its regulatory function is confirmed by reporter gene assays. These data suggest a potentially important role for EU358092 in the aetiology of schizophrenia, either individually or in combination with other genes at this locus.

## 1 Introduction

Chromosome 1p21.3 (chr1:98298371-98581337, GRCh37/hg19) has consistently been associated with schizophrenia by genome-wide association studies (GWAS) (Ripke et al., 2013, Ripke et al., 2011). Efforts to understand the significance of this locus have predominantly focused on the function of one of the genes within this locus, MIR137, and to a lesser extent its neighbouring gene, DPYD (dihydropyrimidine dehydrogenase), which has also been implicated in a range of neurological and psychiatric conditions (Carter et al., 2011, Prasad et al., 2012, Xu et al., 2012). While these genes are the most obvious candidates for causal association, it is important to consider the possibility that there are additional unknown or uncharacterised brain-expressed RNAs at this locus that may also contribute to schizophrenia susceptibility. To address such a possibility, we performed bioinformatic analysis of the locus, using the UCSC Genome Browser (http://genome.ucsc.edu/) to overlay ENCODE (Encyclopaedia of DNA Elements) and GWAS data. In this communication, we identify an RNA termed EU358092, which shares many of the molecular and genetic characteristics previously attributed to MIR137, both in vitro and in vivo. This study extends the potential mechanisms by which the 1p21.3 locus might contribute to schizophrenia risk.

## 2 Methods

### 2.1 Bioinformatic analysis

Bioinformatic analysis was performed with the UCSC Genome Browser, genome build GRCh37/hg19 (http://genome.ucsc.edu; accessed 10/09/2015) and Evolutionary Conserved Region (ECR) browser (http://ecrbrowser.dcode.org; accessed 01/03/2015) to identify ECRs of interest at the MIR137 locus. ECRs were defined as reaching a minimum of 70% homology when the human sequence was compared to other species; this is the default setting of the programme. Schizophrenia genome-wide SNP data from the ‘PGC_SCZ52_may13’ dataset was accessed through Ricopili (http://www.broadinstitute.org/mpg/ricopili/). Aceview, Human 2010 genome (http://www.ncbi.nlm.nih.gov/IEB/Research/Acembly/index.html; accessed 10/09/2015) was used to access RNA-seq data on EU35802 (named ‘jufobu’ in Aceview) from the Non-Human Primate Reference Transcriptome Resource (NHPRTR; http://nhprtr.org/).

LD analysis was performed using SNP genotype data from the CEU/CEPH cohort (European descent) spanning chr1:98,105,779–98,855,147 downloaded from the HapMap Genome Browser (http://hapmap.ncbi.nlm.nih.gov/), release #28. LD analysis was performed using Haploview 4.2 (www.broad.mit.edu/mpg/haploview/) with the following parameters: Hardy-Weinberg *p*-value cut-off, 0.001; minimum genotype cut-off, 75%; maximum number of Mendel errors, 1; minimum minor allele frequency, 0.01) and pair-wise tagging analysis performed (r^2^ threshold, 0.8). Haplotype blocks were determined using 95% confidence intervals (Gabriel et al., 2002).

### 2.2 Plasmid construction

Two ECR domains at EU358092 (termed EU1 and EU2) were cloned into the pGL3-Promoter (pGL3P) luciferase reporter vector (Promega). EU1 and EU2 were amplified by PCR from pooled mixed gender human genomic DNA preparations (Promega) using Phusion High-Fidelity DNA Polymerase (New England Biolabs). Fragments were cloned into the pGL3P vector using Gibson isothermal assembly (NEB Gibson Assembly Master Mix) as described in the manufacturer's protocol, and transformed into XL10-Gold ultracompetent cells (Agilent Technologies) for amplification and purification.

Primers used to amplify each fragment were designed to include 16–20 bp of vector DNA (underlined) flanking the *Sma*I restriction enzyme site for directional cloning into pGL3P. The following primer sets were used:

EU1 ECR primers:

Forward – 5′ AGCTCTTACGCGTGCTAGTGTAGCGAACCAACTGT 3′.

Reverse – 5′ GCAGATCGCAGATCTCGAGTCAAGGCTTATTGTCTTTGG 3′.

EU2 ECR primers:

Forward – 5′ AGCTCTTACGCGTGCTAGAGGCTTCAATGAAAAGAG 3′.

Reverse – 5′ AGATCGCAGATCTCGAGTCATGTGTAATGTCCTGG 3′.

### 2.3 Cell culture and drug treatments

SH-SY5Y neuroblastoma cell line (ATCC number CRL-2266) was maintained in a 1:1 mix of Minimal Essential Medium Eagle (Sigma) and Nutrient Mixture F-12 Ham (Sigma), supplemented with 10% foetal bovine serum (Sigma), 1% penicillin/streptomycin (100 U/ml, 100 mg/ml; Sigma), 1% (v/v) 200 mM l-glutamine (Sigma), and 1% (v/v) 100 mM sodium pyruvate (Sigma). Cells were incubated at 37 °C with 5% CO_2_.

Stock solutions were made for amphetamine, cocaine hydrochloride, lithium chloride, and valproic acid sodium salt (Sigma) using sterile filtered dH_2_O, and diluted as appropriate in SH-SY5Y media. Cells were cultivated for 24 h, before a 1 h incubation with either: vehicle control (sterile filtered dH_2_O), 10 μM amphetamine (Jones and Kauer, 1999, Shyu et al., 2004), 10 μM cocaine (Warburton et al., 2015c), 1 mM lithium (Hing et al., 2012, Roberts et al., 2007) or 5 mM sodium valproate (Pan et al., 2005, Phiel et al., 2001, Zhang et al., 2003). For each drug treatment, *n* = 4. Cells were harvested directly after the 1 h drug treatment for RNA extraction.

### 2.4 Cell culture and luciferase reporter Gene assays

SH-SY5Y cells were seeded at approximately 100,000 cells per well and transfected with 1 μg plasmid DNA and 10 ng pMLuc2 (Novagen) (internal control) using TurboFect (Thermo Scientific). Transfected cells were processed 48 h post-transfection using the Dual-Luciferase Reporter Assay System (Promega). Dual luciferase assays were performed by a Glomax™ 96 Microplate Luminometer (Promega), according to manufacturer's instructions. Fold changes in firefly luciferase activity (normalized to renilla luciferase activity) supported by the EU358092 ECRs over the pGL3P controls were calculated and significance determined using two-tailed *t*-tests. Significance was scored as follows: *P < 0.05, **P < 0.01, ***P < 0.001. For each transfection, *n* = 4.

### 2.5 In vitro RNA extraction

Total RNA was extracted from SH-SY5Y cells using Trizol reagent (Invitrogen) following manufacturer's protocol. The resulting RNA pellets were resuspended in RNase-free water, and 500 ng reverse transcribed into cDNA using the GoScript™ RT system (Promega).

For analysis of gene expression, cDNA was amplified using GoTaq DNA polymerase (Promega) following manufacturer's guidelines, using undiluted cDNA for target genes and a 1:200 dilution for the reference gene.

PCR was performed in a thermocycler with the following conditions: incubation at 95 °C for 5 min, followed by 35 cycles of: (i) 95 °C for 30 s, (ii) 60 °C for 30 s, (iii) 72 °C for 30 s, with a final cycle at 72 °C for 10 min.

Primers used to amplify EU358092:

Forward – 5′ GGTGGGAATTGGGTCTCACA 3′.

Reverse − 5′ GATGAACCTTGACAACGCTGTGTTAAG 3′.

Primers used to amplify actin B control:

Forward – 5′ CACCCCTACAATGAGCTGCGTGTG 3′.

Reverse – 5′ ATAGCACAGCCTGGATAGCAACGTAC 3′.

## 3 Results

### 3.1 Bioinformatic analysis of the MIR137 locus identifies regions of conservation and transcriptional activity

**Fig. 1:**
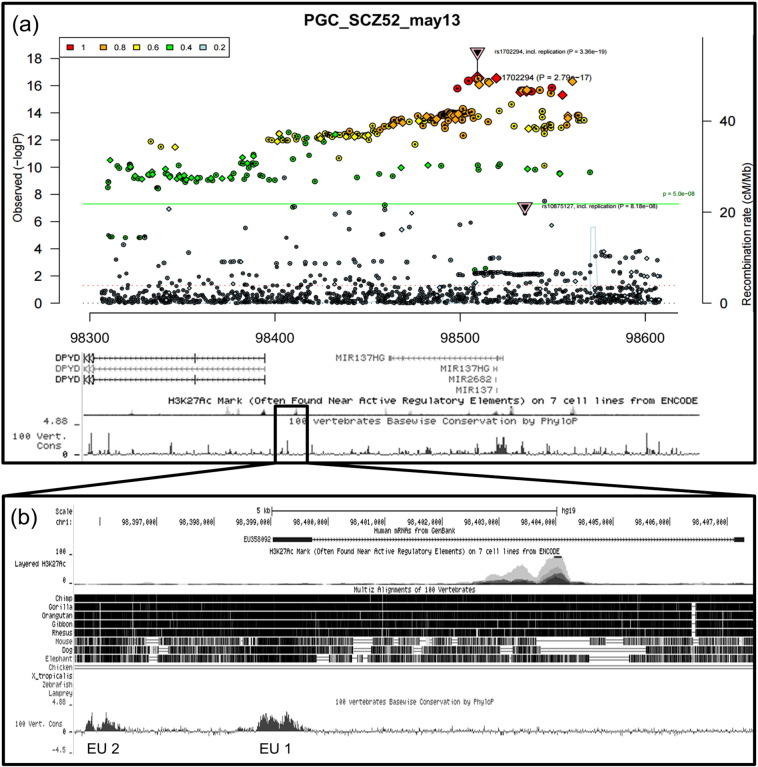
Chromosome 1p21.3 genome wide schizophrenia-associated locus. (a) A composite figure overlaying data across the MIR137 and DPYD genes at chr1:98,300,000–98,600,000 (GRCh37/hg19). The Psychiatric Genomics Consortium's May 2013 schizophrenia GWAS data (PGC SCZ52) was overlaid with H3K27Ac histone marks and evolutionary conservation data from the UCSC Genome Browser. Points above the green line represent GWAS SNPs with a statistically significant *P*-value of 5.0 × 10^− 8^ or more, demonstrating high genome-wide association for schizophrenia across the locus, which expands from MIR137 into EU358092 and the neighbouring gene, DPYD. Evolutionary conservation and H3K27Ac marks over this locus are shown below, and are markers of potential conserved function and active transcriptional regulation, respectively. The region encompassing EU358092 is boxed and expanded in (b). (b) The EU358092 boxed region in (a) is expanded, showing more clearly the peak of H3K27Ac histone modifications, and presenting sequence comparison between multiple species, identifying two evolutionary conserved regions which are displayed as peaks named EU1 and EU2. EU1 overlaps the first exon of EU358092, and EU2 is approximately 2.6 kb upstream of the predicted transcriptional start site of EU358092. The locations of EU1 and EU2 indicate that they may exert regulatory effects on the EU358092 promoter.

**Fig. 2:**
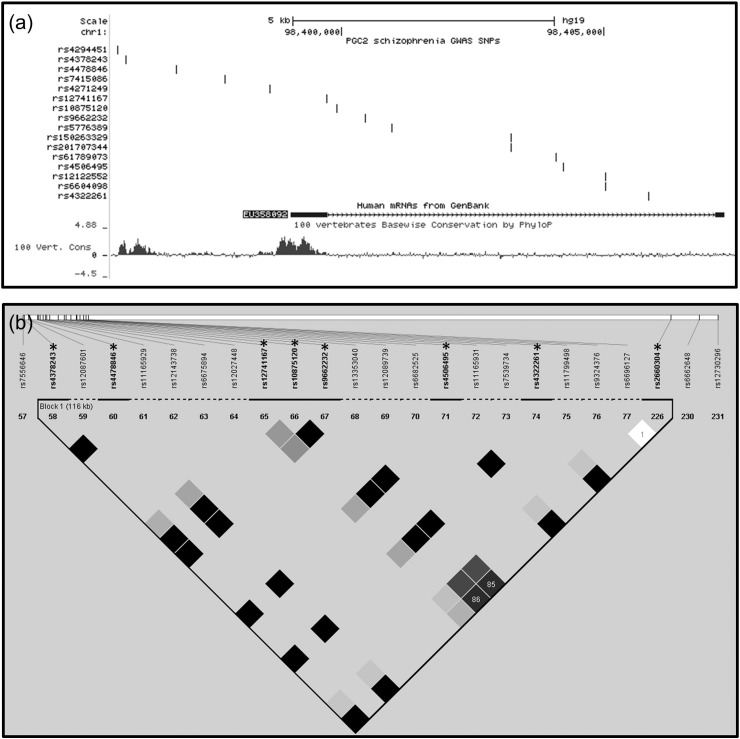
Linkage disequilibrium (LD) analysis of SNPs across the EU358092 locus. (a) Schematic representation of the EU358092 locus and identified ECRs, in relation to 16 schizophrenia GWAS SNPs across this locus. Two schizophrenia GWAS SNPs (rs4294451 and rs4378243) are within EU358092 ECR 2 (EU2), and may therefore modify the function of this conserved element, or tag this as a region of functional interest. (b) LD analysis of SNPs at EU358092 was carried out using SNP genotype data from the HapMap CEU cohort with D′ values. Data for seven of the 16 schizophrenia GWAS SNPs (starred*) was available in this HapMap cohort. The remaining non-GWAS HapMap SNPs were included in the above plot, however the minor allele frequency data for these SNPs was not sufficient to assess LD. All schizophrenia GWAS SNPs at this locus, with the exception of the EU359082 splice site SNP rs12741167, were in complete LD and form a haplotype block as defined by Gabriel et al. (Gabriel et al., 2002). The schizophrenia GWAS SNPs at EU358092 were in LD with the schizophrenia GWAS SNP rs2660304 at the MIR137 internal promoter. The functional significance of the GWAS across this locus may be highlighting either EU358092 or MIR137, which may be acting separately or synergistically to influence the association across this locus with schizophrenia.

Identification of non-coding evolutionary conserved regions (ECRs) at the MIR137 locus is likely to highlight important functional domains, such as transcriptional or post-transcriptional regulatory elements, in addition to other RNAs. Our analysis, using both the UCSC and ECR genome browsers, identified ECRs between DPYD and MIR137 (chr1:98,395,390–98,407,578; GRCh37/hg19) (Fig. 1). These were within the schizophrenia-associated region identified by GWAS (Fig. 1a). Further analysis of this region using GenBank and ENCODE data identified an uncharacterised, brain-expressed transcript named EU358092, overlapping one of the ECRs at this locus, as well as active H3K27Ac histone marks (Fig. 1b) and transcription factor binding sequences (data not shown). In addition, there were schizophrenia GWAS SNPs across the EU358092 RNA sequence (Fig. 2a), and SNPs at this region in LD with schizophrenia GWAS SNPs in the larger locus (Fig. 2b).

### 3.2 Bioinformatic data supports long non coding RNA (lncRNA) status of EU358092

A range of bioinformatic information, accessed through the UCSC Genome Browser, provided support for EU358092 as a lncRNA (long non-coding RNA). GenBank and AceView data listed EU358092 as a human, brain-expressed 869 bp mRNA, having been detected in the amygdala and foetal brain. Current data indicated that this RNA had two exons and covered a genomic distance of 8.27 kb (chr1:98,399,030–98,407,302; GRCh37/hg19) (Fig. 2a). This is consistent with analysis by Derrien et al. on the GENCODE collection of 14,880 lncRNAs which showed that 98% were spliced and 42% were two exon transcripts (Derrien et al., 2012). Analysis of the potential protein coding capacity of the EU358092 sequence by the ExPASy translation tool (http://web.expasy.org/translate/) demonstrated that there are many stop codons in every frame, with the longest open reading frame being 72 amino acids (data not shown).

While MIR137 is clearly an important gene at this locus, EU358092 shares a number of equally important attributes that warrant further investigation. For example, using the UCSC Genome Browser's Multiz 100 vertebrate genome alignment tool, the exons of EU358092 were shown to be conserved to the same degree as the second exon of the MIR137 short transcript, AK311400, exons 3 and 4 of the long MIR137HG transcript, and the second MIR137 locus microRNA, MIR2682 (Fig. 1a). Overall, human EU358092 is 97.2% conserved in the rhesus macaque genome, with 86.1% conservation back to mouse.

### 3.3 GWAS and LD support role for EU358092 in schizophrenia

The Psychiatric Genomics Consortium's 2013 schizophrenia GWAS data (PGC2, available at: https://www.med.unc.edu/pgc) listed 16 schizophrenia GWAS SNPs across the EU358092 transcript and its upstream ECR, as shown in Fig. 2a. Two schizophrenia associated SNPs (rs4294451 and rs4378243) from within the 1p21.3 region were within or directly adjacent to EU358092 ECR 2 (EU2), a potential regulatory element for EU358092. HaploView v4.2 was used to carry out LD analysis of SNPs at the EU358092 locus based on SNP genotype data from the HapMap CEU cohort. Data for seven of the 16 schizophrenia associated SNPs was available in this HapMap cohort, while the remaining non-GWAS HapMap SNPs were included but lacked sufficient data to assess LD (Fig. 2b). Three SNPs at the MIR137 major and internal promoters were also incorporated into this analysis, including the schizophrenia GWAS SNP, rs2660304, which resides at the internal MIR137 promoter, and which we have previously shown to support differential allele-specific expression (Warburton et al., 2015a).

LD analysis showed that six of the seven EU358092 schizophrenia GWAS SNPs tested were in complete LD with each other, and formed a haplotype block which spanned the MIR137 transcript, including complete (D′ = 1) or strong (D′ ≥ 0.85) LD with the MIR137 internal promoter SNP. The remaining schizophrenia associated SNP at this locus with available data, rs12741167, was directly at the annotated splice site of EU358092, and was in partial LD with the other GWAS SNPs at this locus (Fig. 2b). This arrangement of schizophrenia GWAS SNPs in a haplotype block spanning EU358092 and MIR137 may suggest that schizophrenia risk at this locus is mediated through related mechanisms on multiple targets.

### 3.4 Expression of EU358092 in vivo

**Supplementary Fig. 1:**
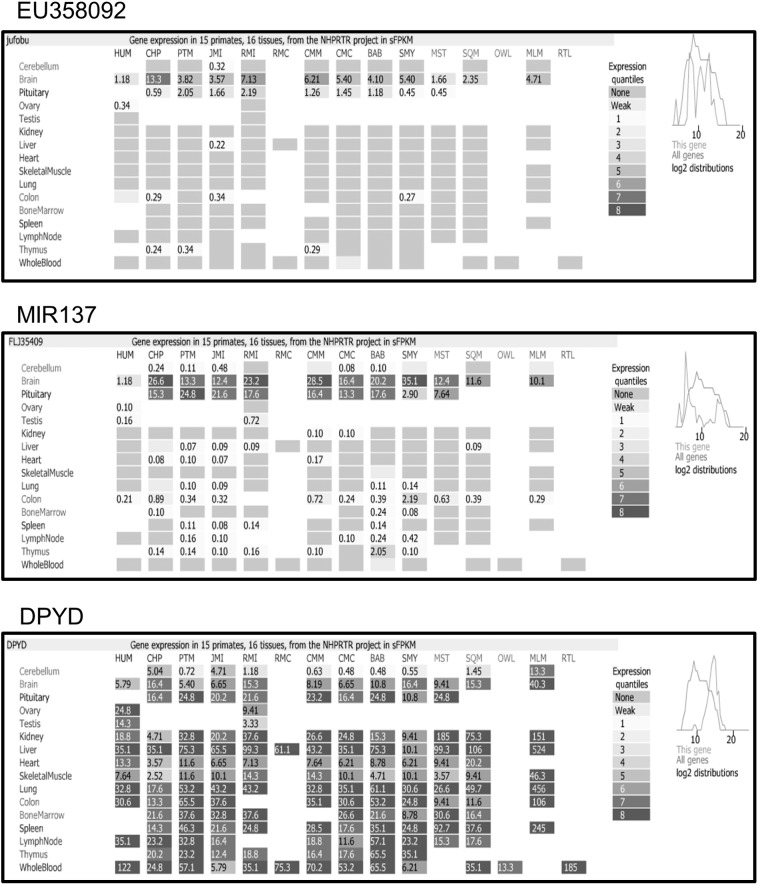
Tissue expression profile of EU358092, MIR137, and DPYD in humans and primates. RNA-seq data from the Non-Human Primate Reference Transcriptome Resource (NHPRTR) comparing EU359082, MIR137, and DPYD expression across 16 tissues in humans and primates. EU359082 is termed ‘jufobu’ in this database. Whilst DPYD is expressed almost ubiquitously across all tissues studied, MIR137 and EU359082 display indistinguishable expression profiles, with their expression being limited to the brain and pituitary in this data set. EU358092 expression was found to be consistently lower than MIR137 across all primate species except humans, where MIR137 and EU358092 expression levels were identical in the human brain. HUM = human; CHP = chimp; PTM = pig-tailed macaque; JMI = Japanese macaque; RM(I/C) = rhesus macaque Indian/Chinese; CM(M/C) = cynomolgus macaque Mauritian/Chinese; BAB = olive baboon; SMY = sooty mangabey; MST = marmoset; SQM = squirrel monkey; OWL = owl monkey; MLM = mouse lemur; RTL = ring-tailed lemur.

Expression of EU358092 in vivo across primate species was compared to MIR137 and DPYD by analysis of RNA-seq data from the Non-Human Primate Reference Transcriptome Resource (NHPRTR, accessed via AceView). EU358092 (termed ‘jufobu’ in this database) displayed a similar expression pattern to that of MIR137, with highest expression restricted to the brain and pituitary gland, whereas DPYD was found to be expressed at high levels across the majority of tissues in all primate species tested (Supplementary Fig. 1).

**Fig. 3:**
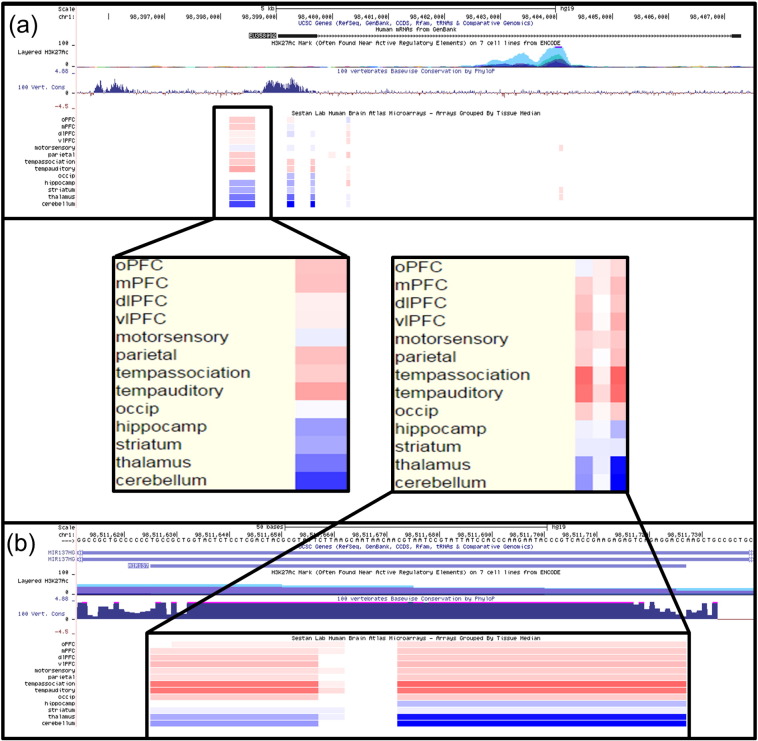
Comparison of MIR137 and EU358092 expression in vivo. (a) Sestan lab brain microarray expression data (accessible through UCSC Genome Browser) for EU358092 showed expression (red) in the orbital and medial prefrontal cortices, as well as the parietal, temporal association and temporal auditory cortices. No expression (blue) of EU358092 was seen in the hippocampus, striatum, thalamus, or cerebellum. (b) Brain microarray data showed MIR137 expression across regions of the prefrontal cortex, including the medial, dorsolateral, and ventrolateral regions, as well as being most highly expressed in the temporal association and temporal auditory cortices. MIR137 was not found to be expressed in the thalamus or cerebellum. The similar expression patterns of EU358092 and MIR137 across brain regions may suggest a co-ordinated pattern of expression for genes at this locus, pointing to a potentially important brain-related function for EU358092.

To further address EU358092 expression specifically in humans, we used UCSC Genome Browser to access the Sestan Lab microarray expression data from four human late mid-foetal brains (Johnson et al., 2009). At this time-point, EU358092 expression was observed in the orbital prefrontal, medial prefrontal, parietal, temporal association and temporal auditory cortices (Fig. 3a). Similarly, brain microarray data showed MIR137 expression across regions of the prefrontal cortex, including the medial, dorsolateral, and ventrolateral regions, as well as being most highly expressed in the temporal association and temporal auditory cortices (Fig. 3b). Neither EU358092 nor MIR137 were found to be expressed in the thalamus or cerebellum. The broadly similar expression patterns of EU358092 and MIR137 may suggest co-ordinated expression of both RNAs, and point to an as yet undetermined brain-related function for EU358092 (Fig. 3).

### 3.5 Activity of EU358092 locus in vitro

**Fig. 4:**
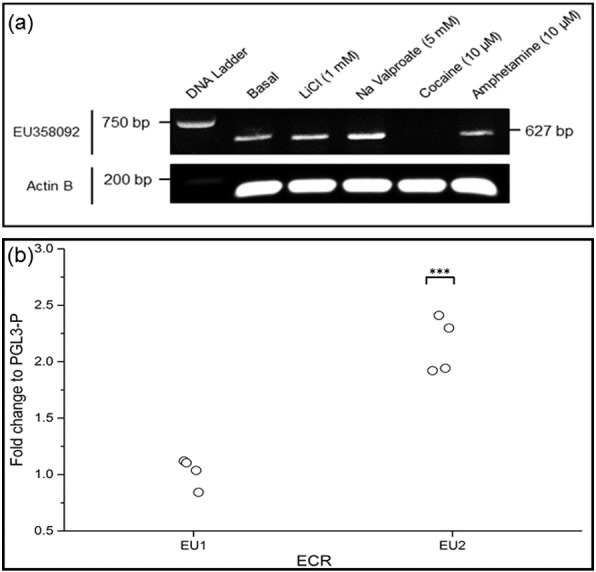
Activity of the EU358092 locus in vitro. (a) Expression of EU358092 was addressed in vitro by PCR of cDNA from SH-SY5Y under basal conditions, and after the following treatments: lithium chloride (1 mM), sodium valproate (5 mM), cocaine (10 μM), and amphetamine (10 μM). Actin B was used as an internal control. EU358092 was shown to be expressed under basal conditions in SH-SY5Y neuroblastoma cells, with expression upregulated following treatment with sodium valproate, and strongly downregulated following cocaine treatment. This pattern of expression in response to challenge was also seen for MIR137 (Warburton et al., 2015b) and may suggest related regulatory responses of transcripts at this locus. (b) Regulatory function of ECR sequences was assessed by dual luciferase assay in the pGL3-Promoter (pGL3P) vector in SH-SY5Y neuroblastoma cells under basal conditions. EU358092 ECR 2 (EU2) supported a 2.12-fold increase in reporter gene expression compared to baseline expression from the empty pGL3P vector, supporting a regulatory role for this sequence, which may act upon the EU358092 promoter to modulate gene expression. EU1 displayed no regulatory function in this assay, which is consistent with this region displaying evolutionary conservation because it encodes the first exon of the EU358092 transcript, rather than being a conserved regulatory element. *N* = 4; ***p < 0.001.

EU358092 expression was confirmed in vitro in SH-SY5Y neuroblastoma cells (Fig. 4a). We have previously demonstrated that expression of MIR137 in this cell line was inducible by drug action (Warburton et al., 2015b). We therefore compared EU358092 expression under similar conditions, after 1 h treatment with lithium chloride (1 mM), sodium valproate (5 mM), cocaine (10 μM), and amphetamine (10 μM) (Fig. 4a). EU358092 was regulated in response to psychoactive compounds, with an increase in expression after 5 mM sodium valproate treatment, and a strong decrease after 10 μM cocaine treatment (Fig. 4a); this was analogous to our previous data on MIR137.

To confirm our hypothesis that the ECR adjacent to EU358092, termed EU2, might have transcriptional regulatory properties, we tested both EU2 and EU1 (which corresponds to exon 1 of EU358092) in reporter gene constructs in SH-SY5Y neuroblastoma cells. EU2, positioned approximately 2.6 kb upstream of the predicted EU358092 transcriptional start site, supported a 2.12-fold increase in reporter gene expression (*p* = 7.97 × 10^− 4^) in the SH-SY5Y cell line (Fig. 4b). This ECR also contained the schizophrenia GWAS SNP rs4378243, with a second schizophrenia GWAS SNP (rs4294451) 25 bp from the boundary of the ECR used in our assay. EU1 had no effect on the baseline expression of the reporter gene (*p* = 0.90). Taken together, these results suggested a regulatory role for EU2, and were consistent with EU1 encoding the first exon of the RNA, rather than constituting a regulatory domain.

## 4 Discussion

GWAS has identified the MIR137/DPYD locus on chromosome 1p21.3 as being strongly associated with schizophrenia. The location of GWAS significant SNPs outside of the MIR137 sequence and protein coding regions of DPYD suggest that a regulatory mechanism might operate at this locus that could result in modulation of expression of these genes. As a microRNA, MIR137 will modulate multiple mRNA targets, with consequences at the protein level. These targets have been postulated to include transcripts from many schizophrenia associated genes, such as CACNA1C, and thus MIR137 has been highlighted as a potential modulator of CNS function that could reveal underlying schizophrenia biology. We have previously used bioinformatic and functional genetics to address differential regulation of MIR137 (Warburton et al., 2015a, Warburton et al., 2015b). In this communication we have used a similar strategy to highlight the potential involvement of the RNA EU358092 in schizophrenia.

We demonstrated that EU358092 was as conserved as MIR137 (Fig. 1) and that the mRNA was tagged by many SNPs significantly associated with schizophrenia by GWAS over the locus (Fig. 2). Analysis of its expression in vivo in the CNS (Figs. 3 and Supplementary Fig. 1) suggested co-expression of EU358092 and MIR137 in many cells. Stimulus inducible expression of EU358092 in response to drug challenge was demonstrated in vitro in SH-SY5Y neuroblastoma cells, which was again similar to previous expression patterns observed for MIR137 (Fig. 4), consistent with both RNAs responding to related transcriptional cues.

EU358092 has many of the characteristics of a non-coding RNA of the lncRNA class. Whilst little is known regarding the function of lncRNAs compared to microRNAs, they are a major class of non-coding transcripts, defined as RNAs of > 200 nucleotides in length which lack protein coding potential. Although only a small fraction (< 2%) of the human genome encodes proteins, the majority of the human genome is capable of being transcribed (Djebali et al., 2012). The 2012 GENCODE v7 release collected 14,880 annotated human lncRNAs (Derrien et al., 2012), yet they remain poorly understood despite their abundance. They often display tissue-specific expression patterns and are thought to function as regulators of gene expression through a broad array of mechanisms (Spadaro et al., 2015, Ulitsky and Bartel, 2013). LncRNAs are increasingly being found to play roles in neurodevelopmental, neurodegenerative, and psychiatric disorders. For example, over 200 differentially expressed lncRNAs have been identified in the brains of individuals with autism spectrum disorder (ASD), which were enriched at genomic loci containing genes linked to neurodevelopment or psychiatric disorders (Ziats and Rennert, 2013). LncRNAs have also been proposed to play a role in schizophrenia, and as such their transcriptional regulation is of paramount importance (Butler et al., 2016, van de Vondervoort et al., 2013). This is consistent with the findings of Liao et al., who demonstrated epigenetic (methylation) differences over lncRNAs in schizophrenia (Liao et al., 2015), and work by Ren et al. which showed modulation of lncRNA networks in early onset schizophrenia (Ren et al., 2015). More specifically, genetic variants of the lncRNA MIAT (also termed Gomafu) have been associated with paranoid schizophrenia in the Han Chinese population (Rao et al., 2015). This lncRNA has been shown to modulate expression of genes involved in schizophrenia such as CSMD1 (Steen et al., 2013) and DISC1 (Barry, 2014). Our study would add another potential lncRNA target for consideration as a modulator of the CNS transcriptome in schizophrenia. Specifically, EU358092 demonstrated many of the characteristics of MIR137 with regard to genetic association and expression in vivo and in vitro. The function of EU359092 was outside the scope of our study, but it should be of interest to those addressing schizophrenia.

The following is the supplementary data related to this article.

## Contributors

John P. Quinn, Vivien J. Bubb, and David A. Collier designed the study. Olympia Gianfrancesco and Alix Warburton undertook experimental work, and Olympia Gianfrancesco wrote the first draft of the manuscript. All authors contributed to and have approved the final manuscript.

## Role of funding source

None.

## Conflict of interest

The authors declare no conflict of interest.
